# The influence of speed abilities and technical skills in early adolescence on adult success in soccer: A long-term prospective analysis using ANOVA and SEM approaches

**DOI:** 10.1371/journal.pone.0182211

**Published:** 2017-08-14

**Authors:** Oliver Höner, Daniel Leyhr, Augustin Kelava

**Affiliations:** 1 Institute of Sports Science, Eberhard Karls University, Tübingen, Germany; 2 Hector Research Institute of Education Sciences and Psychology, Eberhard Karls University, Tübingen, Germany; Katholieke Universiteit Leuven, BELGIUM

## Abstract

Several talent development programs in youth soccer have implemented motor diagnostics measuring performance factors. However, the predictive value of such tests for adult success is a controversial topic in talent research. This prospective cohort study evaluated the long-term predictive value of 1) motor tests and 2) players’ speed abilities (SA) and technical skills (TS) in early adolescence. The sample consisted of 14,178 U12 players from the German talent development program. Five tests (sprint, agility, dribbling, ball control, shooting) were conducted and players’ height, weight as well as relative age were assessed at nationwide diagnostics between 2004 and 2006. In the 2014/15 season, the players were then categorized as professional (*n* = 89), semi-professional (*n* = 913), or non-professional players (*n* = 13,176), indicating their adult performance level (APL). The motor tests’ prognostic relevance was determined using ANOVAs. Players’ future success was predicted by a logistic regression threshold model. This structural equation model comprised a measurement model with the motor tests and two correlated latent factors, SA and TS, with simultaneous consideration for the manifest covariates height, weight and relative age. Each motor predictor and anthropometric characteristic discriminated significantly between the APL (*p* < .001; η^2^ ≤ .02). The threshold model significantly predicted the APL (*R*^*2*^ = 24.8%), and in early adolescence the factor TS (*p* < .001) seems to have a stronger effect on adult performance than SA (*p* < .05). Both approaches (ANOVA, SEM) verified the diagnostics’ predictive validity over a long-term period (≈ 9 years). However, because of the limited effect sizes, the motor tests’ prognostic relevance remains ambiguous. A challenge for future research lies in the integration of different (e.g., person-oriented or multilevel) multivariate approaches that expand beyond the “traditional” topic of single tests’ predictive validity and toward more theoretically founded issues.

## Introduction

Although there are well reasoned warnings in talent research against premature selection in talent identification and development (TID) programs (e.g., [1]), these selections are necessary if a sport association wants to focus its resources on the most talented youth athletes [2]. Thus, the identification of the most promising athletes for the next promotion step is a major task in TID programs.

However, particularly in popular sports, the selection process is a huge challenge, because there are so many talented athletes who compete for only a few positions in adult elite sport. Moreover, talent prognosis seems to be exceedingly difficult in sports with a complex, multi-dimensional performance profile [3]. Soccer possesses both of these attributes. Nevertheless, recent world cup winners (Spain 2010, Germany 2014) obviously benefited vastly from their enhanced engagement in their TID programs, and talent promotion has become a more important and professionalized business in the recent decade. Amongst other measures, several TID programs in soccer e.g., the Elite Player Performance Plan in England [4] have implemented motor performance diagnostics supporting training and / or selection processes. In addition to the holistic evaluations mainly based on talent scouts’ experienced “subjective eye” [5], motor diagnostics and their preferably reliable objective information about future potential of talented players can be of value for clubs and sports associations. Therefore, empirical investigation of motor predictors’ prognostic relevance for long-term success is a key topic in talent research [6, 7].

The association of motor performance in early adolescence with adult success in soccer has been characterized as debatable, and several researchers strongly question the predictive function of motor tests for future success [8–10]. Recent talent research in soccer offers several prospective studies investigating the prognostic validity of talent predictors. This work builds on the fundamental review work by Williams and Franks [11] (see also [12]), who categorized potential personal talent predictors into physical, physiological and psychological factors. (Physiological) speed abilities and (psychomotor) technical skills were amongst the most often-considered predictors and are recognized as particularly important motor factors within soccer associations’ training concepts for TID programs (e.g., [13]) in early adolescence. However, because of a huge variety of study design parameters influencing the research results, *current findings provide an inconsistent picture* with regard to the prognostic validity of motor tests addressing speed abilities and technical skills. Some studies verified the prognostic validity of motor tests [7, 14, 15], whereas others did not find significant associations between test results and later success in youth soccer [16, 17].

Moreover, the use of objective data from motor diagnostics (in particular in terms of reference values) is problematic due to *maturation-related biases in diagnostics* within each age group (e.g., [1, 18]). Talented players’ different maturation statuses and intra-individual developments may cause a weak relationship between juvenile and adult performance that meaningfully decreases the usefulness of motor tests [10]. Thus, for the evaluation of motor predictors’ prognostic validity, maturational characteristics or at least maturation-related characteristics should be considered.

Furthermore, recent studies analyzed motor predictors’ relevance in *prognostic periods* that can be characterized as short (i.e., less than 3 years) or middle (i.e., 3 to 6 years) term. These might be regarded as a limitation of recent research, since some studies questioned juvenile success as an appropriate indicator for success in adulthood [19]. Only a few studies [7, 16, 17] investigating speed abilities or technical skills comprised a prognostic period of more than three years, but even these prospective designs did not last more than 6 years. Thus, there is a lack of research investigating the prognostic value over a long-term period from the beginning of a TID program to its “final destination” (i.e., the transition to the professional level). As TID programs in soccer often start in the U12 age group (e.g., [20]) and the adult level begins at about 19 years of age, the minimum long-term perspective likely needs to be eight years.

Another major problem in talent diagnostics is that complex physiological abilities and technical skills cannot be assessed without *measurement errors*. Until now studies investigating the prognostic relevance of physiological abilities and technical skills in soccer (e.g., [7, 14, 21–23]) have analyzed the predictive validity of single tests (i.e., univariate approaches; e.g., ANOVAs) or the combination of several tests (i.e., multivariate approaches; e.g., MANOVAs or discriminant analysis). As a result, manifest variables are used as indicators, and measurement errors are not considered. However, even highly standardized test procedures cannot avoid limitations regarding reliability (for soccer-specific tests; e.g., [24, 25]). Structural equation modeling (SEM; e.g., [26, 27]) has not been applied in the existing research on talent predictors in soccer. SEM might enable more exact calculations of factors’ prognostic relevance because it considers measurement models (that are capable of estimating the measurement errors) and associations between latent performance factors underlying the motor test results.

### The present study

This study pursued two objectives with a focus on speed abilities and technical skills while also considering anthropometric and relative age characteristics. *First*, the predictive validity of motor tests, anthropometric variables, and relative age were investigated over a long-term period from shortly following the entrance into the German TID program (U12) until after the transition into adult soccer (> U19). *Second*, this study analyzed the long-term predictive power of the two theoretical (latent) performance factors (i.e., speed and technique) with simultaneous consideration of maturation-related characteristics such as relative age and anthropometric variables.

## Methods

### Sample and design

**Fig 1:**
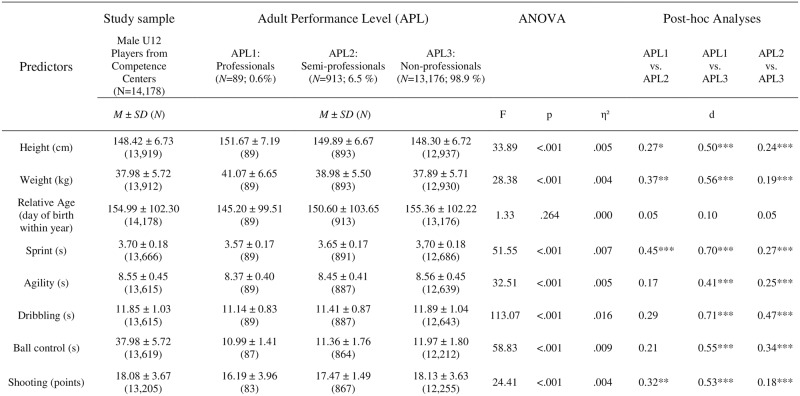
Descriptive and inferential statistics for the diagnostics in U12 separated by players’ adult performance level. *p < .05, **p < .01, ***p < .001. The motor tests (and relative age) are negatively coded, i.e. lower values indicate better performances (or an older age) for these predictors.

This *prospective cohort study* investigated the data from *N* = 14,178 male competence centers athletes (11.32 ± 0.28 years old) who belong to the top 4% of the boys playing soccer at this age [28]. All individuals participated in motor diagnostics—which were implemented nationwide in the German TID program in 2004 (see [29])–in the U12 age group in the fall of 2004, 2005 or 2006. The U12 motor tests’ results served as predictors for players’ later success at the adult level in the 2014/15 soccer season leading to a prognostic period of eight, nine or ten years, respectively. The players’ height, weight, and relative age were considered as covariates (descriptive sample characteristics are presented in Fig 1).

Before entering the TID program, players’ parents provided written informed consent for the recording and scientific use of the data collected in the motor diagnostics. DFB staff members (coaches with at least UEFA B-License) conducted the motor tests and assessed the predictor variables of the present study. The DFB provided the authors with players’ data of three birth cohorts (1993, 1994, and 1995). The university’s ethics department and the scientific board of the DFB approved the implementation of this study.

### Measures

#### Predictors

Before performing the motor tests, each player’s *height*, *weight*, and *relative age* (measured by the day of birth within a calendar year) were registered. The motor test battery, consisted of five tests that were all negatively coded, so that a lower value indicated a better performance. Players were tested in *sprint* (time for a 20 m linear sprint), *agility* and *dribbling* (time for a slalom course without and with a ball, respectively), *ball control* (time for six passes against two opposing impact walls), and *shooting* (8 shots at three different target fields rated by a coach with regard to precision and speed). Times for sprint, agility and dribbling were measured utilizing light barrier systems whereas times for ball control were assessed with chronographs. While two attempts were conducted for each of these tests only the better one was counted. Players were provided with ample time to recover between the attempts.

Höner et al. [30] provided a more detailed description of the individual tests and analyzed the test battery’s psychometric properties for a sample consisting of nearly 70,000 U12 to U15 competence center players. They found excellent internal consistencies in terms of Cronbach’s Alpha for sprint (α = .95) and agility (α = .91), whereas the values for the instrumental reliability of the more football-specific tests, ball control (α = .68), dribbling (α = .61) and in particular shooting (α = .41), were considerably lower. Furthermore, this study analyzed the underlying performance factors of the motor test battery and explored two factors—i.e. *speed abilities (SA)* and *technical skills (TS)—*with an exploratory factor analysis [30].

In line with these findings concerning the factorial structure, the present prospective cohort study proposed a measurement model consisting of two latent factors determining the performance in the single motor tests. Thereby, sprint and agility were restricted to load on the SA-factor, and ball control as well as shooting on the TS-factor. Because dribbling includes a technical and a speed-related component, this test was part of both factors. A confirmatory factor analysis confirmed this structure including the five motor predictors sprint, agility, dribbling, ball control and shooting with the data of the present cohort study (χ^2^(3) = 34.657, *p* < .001, RMSEA = .027, CFI = .995, TFI = .983).

#### Criterion

The *adult performance level (APL)* in the 2014/15 season was utilized to quantify players’ success in adulthood as criterion variable. More specifically, the rosters of the first five German leagues regarding the players’ names and birth dates were examined [31, 32]. Players who participated in the U12 motor diagnostics and played at minimum one match in the 2014/15 season in one of the five highest leagues in Germany were identified. Based on their placement in the various leagues, they were then assigned to the professional (APL1), semi-professional (APL2) or the non-professional level (APL3). APL1 players appeared in one of the first three German leagues (Bundesliga, 2. Bundesliga, 3. Liga). Players from the fourth and fifth division (Regionalliga, Oberliga) were appointed to the semi-professional level, APL2. Players who did not reach one of these leagues were classified as non-professionals (APL3). Less than 1% of the players were assigned to the professional level (*n* = 89; 0.6% of the players in the examined sample), whereas *n* = 913 players (6.5%) joined the semi-professional level and *n* = 13,167 the non-professional level (92.9%).

### Statistical analysis

Data were analyzed using SPSS version 22 and Mplus version 7.1. With regard to the *prognostic relevance of the motor diagnostics*, the significance of mean differences between players achieving different APLs in the 2014/2015 season was determined by a one-way MANOVA including the five single motor tests as dependent variables (*objective 1*). Additionally, a corresponding MANCOVA was conducted incorporating height, weight and relative age as maturation-related covariates.

In order to provide robust results regarding the predictors’ prognostic relevance, the dataset of three birth cohorts (1993, 1994, 1995) was accumulated, so that the sample of the highest selection levels (APL1) achieved a sufficient number. However, the cohorts may confound the analysis of motor performances’ differences between selection levels [33]. To investigate this assumption, a two-way MANOVA was conducted testing whether there was an interaction effect of APL x cohort on the five motor tests. Because of the non-significant interaction (*F*(24; 48,328) = 0.96, *p* = .51) and non-significant differences for players from different birth cohorts with regard to height (*F*(2; 13,616) = 0.31, *p* = .74), weight (*F*(2; 13,909) = 2.43, *p* = .09), as well as relative age (*F*(2; 14,175) = 1.33, *p* = .27), the cohort variable was not further considered as a confounder in the following analysis.

To gain insight into the *prognostic relevance of the single predictors* (five motor tests, three maturation-related characteristics), univariate ANOVAs were conducted analyzing mean differences between the different performance levels for each predictor. Post hoc tests (Tukey HSD) were conducted to contrast the three APLs within multiple group comparisons. Effect sizes were provided independently from their significance, as the group sample sizes for the three APLs differed strongly. Cohen’s *d* and η^2^ were calculated as effect sizes and classified in accordance to [34]. In this process, Cohen’s *d* was computed as the mean difference divided by the pooled standard deviation:

$$d=\frac{M_{1}-M_{2}}{\sqrt{\frac{\left(n_{1}\;–\;1\right)\;*\;SD_{1}^{2}\;+\;\left(n_{2}\;–\;1\right)\;*\;SD_{2}^{2}}{n_{1}\;+\;n_{2}\;–\;2}\;}}.$$

Additionally, players’ future success was predicted in a logistic regression model by the two correlated latent performance factors SA and TS with simultaneous consideration of players’ height, weight and relative age that served as manifest covariates (*objective 2*). Thereby, the prediction of APL—as a criterion variable with ordered categorical outcome levels—was based on a threshold model with an underlying continuous latent response variable (for more information, see [35, 36]). For a clearer view of a player’s relative chances depending on several predictors, odds ratios (for non-standardized variables) were computed as effect sizes. In accordance to Muthén [37], *R*^*2*^ was examined to quantify the amount of variance explained by the logistic regression model and refers to the explained variance proportion in the underlying continuous latent response variable.

## Results

### Predictive value of motor tests and maturation-related characteristics (objective 1)

**Fig 2:**
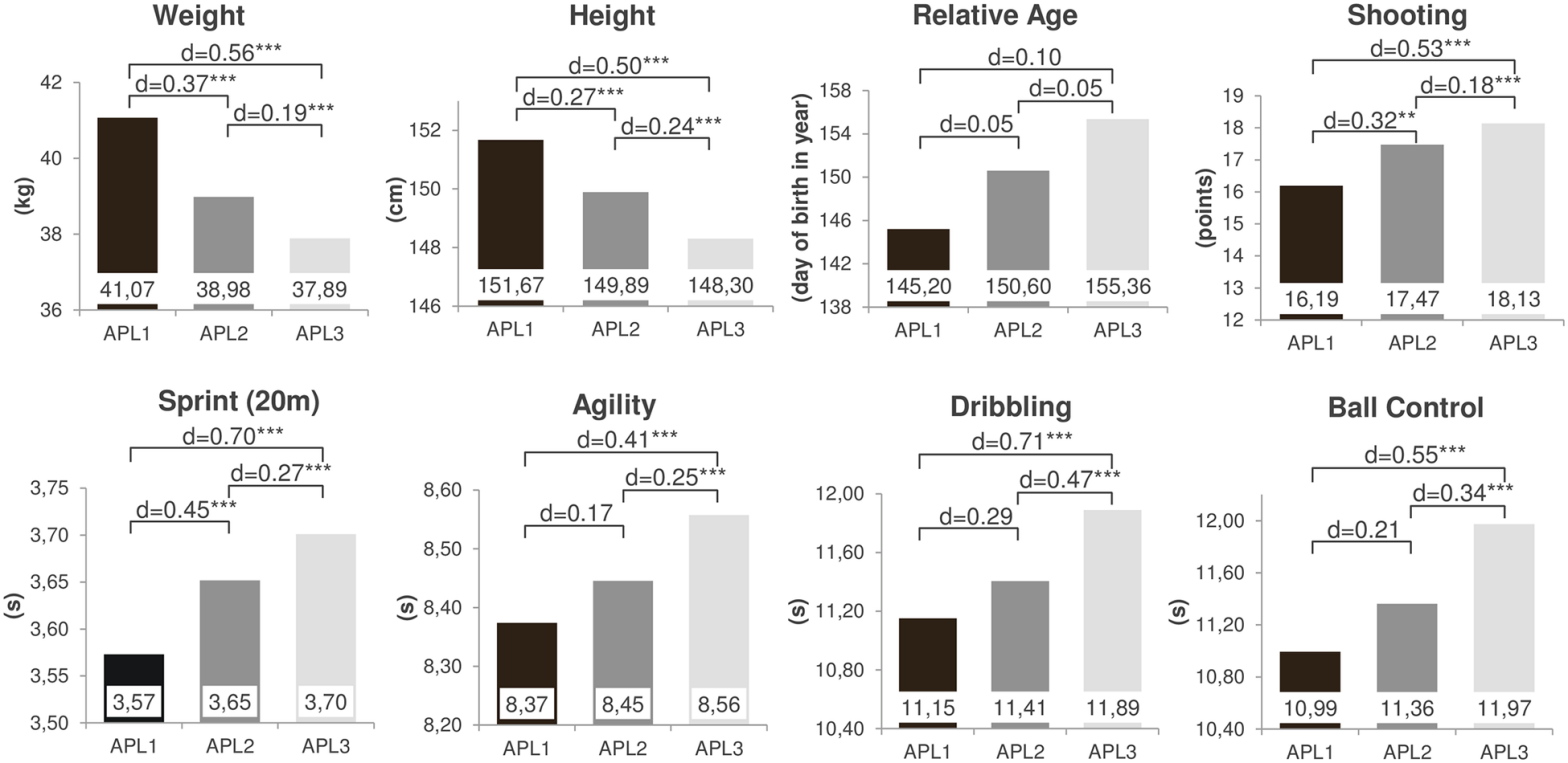
Descriptive statistics for the diagnostics in U12 separated by players’ adult performance level (Including the effect sizes for the multiple group comparisons, see also Fig 1). *p < .05, **p < .01, ***p < .001

The U12 results for all predictors (i.e., the motor performance tests, the anthropometric characteristics and relative age) correspond to the later achieved APL (Fig 2). Players who reached APL1 performed better in all motor test than the players who made it to the semi-professional or non-professional level. The same holds true for players who became semi-professional in comparison to non-professional players. Additionally, U12 players who reached APL1 were the tallest, heaviest and slightly oldest. This also extends to the APL2 players as compared to future APL3 players.

The *motor diagnostics’ prognostic relevance* was verified by significant mean differences concerning the three APLs (*F*(10; 24,174) = 31.48, *p* < .001, η^2^ = .01) and this APL effect remained significant in the MANCOVA under consideration of the maturation-related covariates (*F*(10; 23,902) = 33.40, *p* < .001, η^2^ = .01). ANOVAs demonstrated the significance for the five single motor tests and for the anthropometric variables height and weight (each *p* < .001; Fig 1). Only relative age failed to reach significance (*F* (2; 14,175) = 1.33, *p* = .26). However, although the motor and anthropometric variables showed a prognostic relevance for future success in adulthood, effect sizes were rather small (each η^2^ < .02).

The *multiple group comparisons* revealed significant mean differences in all considered variables (except for relative age) between APL3 and the two higher levels (Figs 1 and 2). Both, future professional and semi-professional players performed better in the U12 diagnostics than the non-professionals. The effect sizes in the motor test results ranged from small (e.g., a non-significant *d* = 0.17 between APL1 and APL2 in agility or *d* = 0.18 between APL2 and APL3 in shooting) to medium effect sizes (e.g., *d* = 0.71 and *d* = 0.70 between APL1 and APL3 in dribbling and sprint, respectively). Moreover, significant mean differences between APL1 and APL2 could be found for shooting (*d* = 0.32) and sprint (*d* = 0.45), showing the most discriminative power between the two highest levels. Although the dribbling test was able to differentiate both, between APL3 and APL1 (*d* = 0.71) as well as between APL3 and APL2 (*d* = 0.47), this test narrowly failed to reach significance in separating between APL1 and APL2 (*d* = 0.29, *p* = .07).

With regard to the *anthropometric variables* height and weight, multiple group comparisons revealed significant differences between all the examined APLs. The effect sizes ranged from *d* = 0.19 (APL2 vs. APL3 regarding weight) to *d* = 0.56 (APL1 vs. APL3 regarding weight). In accordance to the non-significant overall comparison, the relative age of the U12 competence center players was a less meaningful predictor for future APLs (*d* ≤ 0.10).

### Predictive value of latent performance factors (objective 2)

The complex logistic regression model considered the maturation-related characteristics as covariates and predicted APL by the two factors SA and TS. The analysis of the model yielded *R*^*2*^ = .25, indicating that 25% of the variance in APL could be explained by the model.

**Fig 3:**
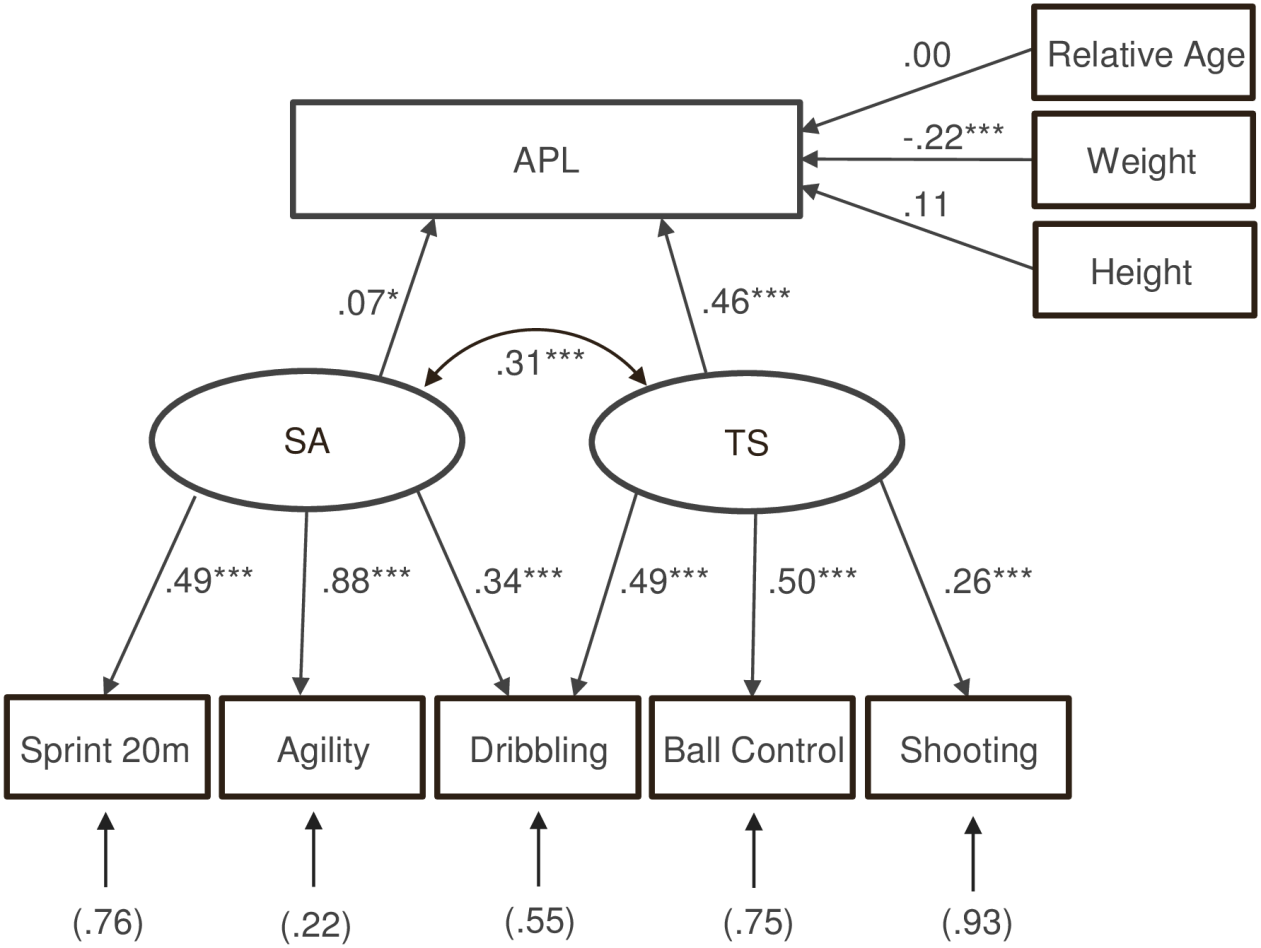
Players’ adult performance level predicted by two latent factors (SA and TS) with simultaneous consideration of maturation-related characteristics as covariates. *p < .05; **p < .01; ***p < .001. The regression coefficient for relative age was estimated as β = -.003.

Fig 3 presents the factor loadings and regression coefficients of the logistic regression model. First, the manifest variables’ single loadings on the latent variables confirmed the preassigned factorial structure of the two latent factor variables SA and TS. All single tests loaded distinctively on their respective factor (.26 ≤ λ ≤ .88; each *p* < .001). Furthermore, the factor loadings for the dribbling test confirmed that SA (λ = .34) as well as TS (λ = .49) are the underlying performance factors.

The logistic regression model revealed a significant influence on APL for the latent factor variable SA (β = .07, *p* < .05) and TS (β = .46, *p* < .001). In this case, a notable correlation (*r* = .33) between SA and TS leads to a rather small contribution of SA to APL. Nevertheless, the technical components represented by TS possess an even more incremental predictive power compared to SA containing predominantly speed-related abilities. With regard to the two latent factor variables SA and TS, the ORs e^β^ of the logistic regression model (*OR*_*SA*_ = 4.58, *OR*_*TS*_ = 6.72) indicated that a one standard deviation (*SD*_*SA*_ = 0.09, *SD*_*TS*_ = 0.50) increase in the respective latent factor variable improved the chances of achieving the professional level by a factor of 1.15 (= 4.58^0.09^) for SA and by a factor of 2.59 (= 6.72^0.50^) for TS, respectively.

Furthermore, a significant impact of players’ weight was verified by the model, indicating that heavier U12 players had higher chances of achieving a better APL (β = -.22, *p* < .001). In addition to this, height (*p* = .32) and relative age (*p* = .84) revealed no further predictive power.

## Discussion

Compared to recent studies investigating the prognostic relevance of talent predictors in youth soccer, this study assessed a large sample over a long-term period from the beginning of a talent development program in early adolescence to the entrance into an adult career. Dealing with the claim for a multidimensional approach, this work addressed examples of physiological (e.g., sprint), psychomotor (e.g., ball control) and physical (e.g., height) predictors. Besides the well-established procedures analyzing manifest variables based on the general linear model (e.g., ANOVAs), this study also used SEM (here a logistic regression threshold model) as a further multivariate approach allowing for the consideration of latent performance factors as well as their interdependent relations. Both approaches verified the prognostic validity of the five motor tests and the anthropometric variables over a long-term period (≈ 9 years). Only the relative age did not discriminate between the future achieved performance levels within the study sample.

### Predictive value of motor tests and maturation-related characteristics (objective 1)

For interpretation of the *prognostic relevance*, one must consider that design features strongly affect the estimates of predictors’ prognostic relevance. For example, the duration of the investigated prognostic period and its relative developmental phase play an important role, because discriminating factors among youth players vary between the different development stages of adolescence [38]. In addition, the sample size (determining the statistical test power) and the performance levels at which the predictors and criteria were assessed (leading to different “ranges of talent”, [39]) are further potential moderators of the effect size.

Because of the strong influence of design features, the current results are best comparable with findings from Höner and Votteler [7]. That study also analyzed the prognostic relevance of motor diagnostics within the German soccer TID in early adolescence, but for the cohorts 1993 to 1997 and over a mid-term period from the onset of puberty to middle-to-late adolescence (including the transition from basic to elite talent promotion). Future success was operationalized with four performance levels in which the highest (youth national team) and the lowest level (not further selected for the TID in U16-U19) were most similar to APL1 and APL3 in the present study. The present results confirm the former mid-term prognostic results for an even longer period that additionally includes the entrance into adult soccer as a further crucial career transition. Both studies detected similar effect sizes. Multiple group comparisons between the highest and lowest performance levels revealed the highest effect sizes for sprint (*d* = 0.67 and *d* = 0.70 for the mid- and long-term study, respectively) and dribbling (*d* = 0.64, *d* = 0.71). Furthermore, the studies found medium effect sizes for agility (*d* = 0.45, *d* = 0.41) and ball control (*d* = 0.59, *d* = 0.54). However, with regard to the shooting test, the effect size in the present study (*d* = 0.53) was considerably higher than in the mid-term study (*d* = 0.30). This is quite remarkable given the longer period, as one would assume that, all else unchanged, the association between the predictor and the criterion variable decreases over a longer prognostic period.

For reasons of comparability in the following paragraphs, the results from other studies that refer as much as possible to the age group U12 are reported. Moreover, the present study’s differing effects between APL1 and APL3 are compared to similar findings from the other studies (e.g., youth national team vs. non-selected). Effect sizes were translated in terms of Cohen’s *d* on the basis of the descriptive statistics provided by the original studies (in cases where a study did not provide Cohen’s *d* as an effect size).

Regarding the prognostic relevance of *speed related predictors*, this study revealed slightly larger effect sizes for sprint and nearly identical effect sizes for agility compared to other prospective studies dealing with players at this age. Whereas Le Gall et al. [17] found no prognostic relevance for the achieved adult performance level of French U14 academy players, Gonaus and Müller [6] revealed significant small to medium effect sizes for sprint (*d* = 0.36) and agility (*d* = 0.49) in their 3-year prospective study with Austrian U15 youth academy players. In their 2-year study with elite Belgian U12 players, Deprez et al. [21] found small to medium effect sizes for their tests in 5 m sprint, 30 m sprint and agility (*d* = 0.3, *d* = 0.6 and *d* = 0.5, respectively), where only the effect for the 30 m sprint reached significance. Further studies [14, 40–42] confirmed the prognostic validity of speed and agility tests. However, various design parameters of these studies differed from the presented study (e.g., other performance criteria, lower performance level), so that detailed comparisons of the effect sizes become superfluous.

Compared to the research of speed related predictors, there are considerably fewer prospective studies covering the prognostic relevance of *technical skills* [25]. In this regard the observed medium effect sizes for dribbling, ball control, and shooting fit into the broad range of effect sizes found in previous research. In their 2-year prospective studies, Figueiredo et al. [14] and Deprez et al. [21] analyzed football-specific technical skills’ prognostic relevance in the age group U12. Performances in a wall pass and shooting accuracy test were non-significant predictors regarding the prospective playing status of Portuguese regional club players. By contrast, a ball control test (*d* = 1.08) and a dribbling test (*d* = 1.20) showed prognostic relevance in the same study [14]. To a smaller extent, dribbling was also a significant predictor (*d* = 0.3) for Belgian elite players’ future playing status [21]. Furthermore, albeit for older Dutch youth academy players in late adolescence (U16-U19), Hujigen et al. [20] detected medium effect sizes indicating lower performances in different dribbling features for players who were deselected from the youth academies one year later. Whereas the prognostic effects reached significance for the peak shuttle dribble (*d* = 0.66) as well as for the repeated shuttle dribble test (*d* = 0.60), they failed significance for the slalom dribble test (*d* = 0.37).

The present results emphasize the importance of the *anthropometric variables* height (*d* = 0.50) and weight (*d* = 0.55) in early adolescence for more or less successful adult players. These medium effect sizes are quite similar to the prognostic value displayed by Gil et al. [40] for weight (*d* = 0.41), but also lower than the prognostic relevancies for height reported by Figueiredo et al. [14] (e.g., *d* = 1.03, in U12/U13) and Gil et al. [40] (e.g., *d* = 0.81, in U15) in their studies with regional club players (prognostic periods ≤ 2 years). However, they are higher than the effects of the prospective studies conducted by Deprez et al. [21], Gil et al. [41], Gravina et al. [42] and Le Gall et al. [17], who all found non-significant impact of these variables in early adolescence for future success.

Still, it remains unanswered whether differences in the anthropometric variables are caused by maturation-related biases in the talent selection (e.g., biologically more mature players are more often selected) or whether these variables have to be considered as performance factors in adulthood. Indeed, the 89 APL1 players were, as adults, on average 182.20 cm tall (based on the data provided by [31]). Thus, the “successful” group of this study was slightly taller than the corresponding German population of 18 to 25 year-old males who are on average 181 cm tall [43]. As the difference between the APL1 and APL3 players in the U12 age (3.37 cm) was noticeably larger, this might indicate an advanced biological maturation for future APL1 players in the U12 age group. However, for clarification of this important issue, more sophisticated and valid measures for biological maturity (e.g., skeletal age or ages at peak height velocity; [44]), differentiated analyses regarding the influence of anthropometric variables and playing position, (e.g., [45]) and a repeated measurement lasting over different developmental phases would be necessary.

Several causes might have led to the non-significant influence of *relative age*. The main reason is probably that the U12 players who participated in this study were already selected for the German TID program. These players ranked among the top 4% of their age cohort and the influence of relative age on performance factors is reduced in already selected samples [18]. Thus, a relative age effect already existed within this study sample (i.e., 60.4% of players were born in the first half of the year). The rate of the APL1 players (61.8%) is quite comparable, leading to non-significant effect sizes in the multiple group comparisons for relative age. However, the fact that the extent of the relative age effect at the beginning of a TID program is similar to its magnitude in adult professional soccer does not imply that the relative age effect is irrelevant for the TID process. For example, there is a considerably higher relative age effect in middle-to-late adolescence. More than 70% of the players that are selected for German youth academies and youth national teams are born in the first half of the year (for similar data in France, Spain or Switzerland, see [46–48]). Upon entrance into the adult professional level the relative age effect is reduced (but still present), because at this point previously selected relatively younger players have even higher chances of becoming a professional [49].

### Predictive value of latent performance factors (objective 2)

Overall, the ANOVA analyses with manifest variables indicate a small magnitude of explained variance regarding the three adult performance levels (η^2^ < .02). However, despite the small explained variance, the quite relevant effect sizes derived by the multiple group comparisons are a first hint that the tests obtained prognostic relevance. The results of the SEM confirm these general findings of the “traditional” approaches, but also go beyond these findings with regard to the 1) overall explained variance, 2) role of the covariates, 3) consideration of the underlying performance factors and 4) attributes of the measurement model.

*First*, the logistic regression model led to a considerably higher magnitude of explained variance (*R*^*2*^ = 25%), so that SA and TS explain with simultaneous consideration of anthropometric characteristics and relative age a quarter of the APLs. To the authors’ knowledge no study in soccer exists using a latent variable approach for the estimation of the prognostic relevance of performance factors. Thus, the results for objective 2 cannot be compared to other studies. Nevertheless, there are two main reasons for the increase in explained variance in the latent variable model. One, the latent variable model corrects for attenuation due to measurement error of single manifest variables (e.g., [26]). The correction of attenuation is conducted by including several indicators of the latent exogenous TS and SA factors. Within the latent structural model, regression coefficients are unbiased and error free. Two, the manifest discrete outcome variable with its levels (APL1-3) is approximated by an underlying latent continuous variable y*, which is linked by a threshold model to the observable outcome y. The underlying latent continuous variable y* represents a more realistic “true ability” factor which leads to the manifest outcome via a threshold parametrization (for technical details of the Mplus latent variable framework see [50]). As a result of these two advances, the latent variable model approach better explains the relationship between the variables.

*Second*, regarding the role of the covariates, only weight (β = -.22, *p* < .001) was a significant anthropometric covariate, indicating that heavier players have a greater chance of achieving a higher APL. Furthermore, height (β = .11, *p* = .32) provides no further explanation of the APL and the relative age effect had no additional influence on the APL. However, due to the strong association of weight and height (*r* = .73), it would obviously be wrong to conclude that height in early adolescence is unimportant for future success. According to the results for objective 1, the prognostic relevance of the two anthropometric variables was quite similar. However, the insights into the impact of maturity characteristics on the TID process are limited in this study, as its focus laid on the relevance of speed and technical performance factors with simultaneous consideration of maturation-related covariates. Therefore, the anthropometric variables and relative age were entered on the same level within the logistic regression as equal covariates and no underlying latent maturity factor was integrated into the model nor was height conceptualized as a predictor for weight (that then might have served as a mediator on APL). For a more concrete clarification of the impact of maturity characteristics on the TID process more valid measures for maturity, repeated measurements and a theoretical model including core assumptions of the mechanisms would be necessary.

*Third*, the analyses revealed significant path coefficients for both underlying performance factors (β = .07, *p* < .05 for SA; β = .46, *p* < .001 for TS). It was observed that TS had a stronger effect than SA leading to the interpretation that technical skills seem to be stronger predictors than speed abilities. However, some parts of the difference between the effects might be due to the collinearity between TS and SA, which means that TS and SA have variance in common. Therefore, the difference in the effects should be interpreted cautiously.

*Fourth*, the attributes of the measurement model validated the particular role of dribbling as it was influenced by both theoretical factors (λ = .34 for SA and λ = .49 for TS). The reason for this is that speed-related as well as technical skills impact dribbling performance in this test. The TS-factor influences the dribbling and ball control performances to nearly the same relevant extent (λ ≈ .50). The considerably lower coefficient on shooting (λ = .26) is attributable to a high proportion of measurement errors, because of the shooting test’s low reliability. Regarding the SA-factor loadings on the indicators sprint (λ = .49), agility (λ = .88) and dribbling (λ = .49), this study confirms the factor solution found by Höner et al. [30] with data from other cohorts (1999, 2000) from the age group U12 in German soccer TID (with factor loadings λ = .50, λ = .92, and λ = .35 for sprint, agility and dribbling, respectively). The SA-factor mainly affects agility, whereas the sprint performance seems to be influenced by other factors as well. In accordance with this, the estimated variances of the measurement errors (ϴ^δ^ = .76 for 20 m sprint and ϴ^δ^ = .22 for agility) illustrate that in particular agility performance is explained by the SA-factor (ca. 78%). As both tests have similar (excellent) reliabilities, these differences in the factor loadings and variance of the measurement errors are not caused by different measurement errors. Confirming the findings from Höner et al. [30] the impact of the SA-factor on the dribbling performance is lower. This is caused by the additional influence of the TS-factor as well as by the lower reliability of this football-specific test compared to the sprint and agility test.

## Conclusion

The study provides reliable empirical knowledge on the prognostic relevance of speed related and technical skill tests in a realistic setting (i.e., a nationwide census assessment within a concrete TID program). The results demonstrated motor predictors’ prognostic validity over a long-term period (≈ 9 years), even after controlling for maturation-related characteristics. However, the sensitivity of such tests is by far too low to apply them as exclusive and reliable information for selection decisions in a TID program even though repeated measurements as well as further predictors—e.g., personality [51] or cognitive performance [52] factors—may increase the explainable variance in future performance.

Thus, identifying talent in soccer still remains a very complex (practical and theoretical) problem, and multidimensional approaches are recommended [38] as they may prove more successful in talent identification [3]. With a focus on practical utility of motor tests, recent studies followed a multivariate approach mainly by combining manifest variables within any specification of the general linear model. In addition, this study applied SEM to consider as well the underlying latent performance factors, providing further insights into the role of speed abilities and technical skills for talent identification. The latent variable model has a greater predictive power than the ANOVA approach and technical skills in early adolescence seem to have a stronger effect on future performance than speed abilities.

A promising prospect and likewise a huge challenge for future talent research lies in the application and integration of different multivariate approaches in order to go beyond the “traditional” topic of single tests’ predictive validity and to be able to work on more theoretically founded questions. One step in this direction was undertaken with this study. Other promising methodological approaches (which until now have dealt with other topics of talent research in soccer or similar sports)–such as person-oriented pattern analyses [15], mediator analysis based on SEM [53], multilevel modelling of (quasi-)longitudinal data [23, 54, 55], evolutionary analyses of individual success [25], or higher-dimensional models based on singular value decomposition and receiver operating characteristic analysis [56]–should be further examined and compared with each other to identify the corresponding strengths and weaknesses. In doing so the research on talent may develop in both directions, providing coaches with more scientifically sound tools for supporting their TID process (practice-orientated perspective) as well as offering a deeper understanding of the underlying factors of a talented player and his or her positive development (science-oriented perspective).

## Supporting information

S1 File Dataset. (TXT)

S2 File Related manuscript item. (PDF)
