# Supplementary material for: The influence of speed abilities and technical skills in early adolescence on adult success in soccer: A long-term prospective analysis using ANOVA and SEM approaches
Source: PLoS One. 2017 Aug 14;12(8):e0182211. doi: 10.1371/journal.pone.0182211 (PMC5555567; doi:10.1371/journal.pone.0182211)
Supplement: S2 File — (PDF) [file pone.0182211.s002.pdf]

To decrease the possible influence of the economic development, transportation and stadiums facilities, standard of living, interest and acceptance of football on the spectators' attendance, only last five world cups (past 20 years) were followed. The magnitude of correlation suggested by Hopkins was used.
RESULTS: The means of spectators' number, temperature, relative humidity and heat index during the last five Football world cup are 3178856 ± 319917 spectators, 18 ± 3.18, 70 C° ± 13.44 and 17 % ± 3.42, respectively. **Negative correlations were observe between the spectators' number and various weather variables (i.e., temperature, relative humidity and heat index). None of these correlations was statistically significant. Trivial and small corrections were observed between spectators' number and both temperature and heat index, respectively. A trend of significant correlation was observed between the spectators' number and the relative humidity. Despite non-significant, this correlation is considered meaningful (large).** Negative and non-significant correlation was shown between the weather variables and the spectators' attendance. "Trivial" correlation was shown between the temperature and the spectators' attendance. A "Small" correlation was observed between the heat index and the spectators' attendance.
CONCLUSIONS: The extreme summer weather conditions in Qatar would affect the spectator numbers and thus the decision taken by FIFA to change the host time to December seems to be appropriate.

2798 Board #321 June 3, 11:00 AM - 12:30 PM

Prognostic Relevance of Motor Predictors in Early Adolescence for Reaching Professional Soccer Level in Adulthood

Oliver Höner, Augustin Kelava, Daniel Leyhr. *University of Tübingen, Tübingen, Germany.*
Email: oliver.hoener@uni-tuebingen.de
(No relationships reported)

Several talent development programs in soccer have implemented diagnostics measuring motor performance factors. However, there is debate in the field of talent research about the prognostic relevance of such diagnostics in early adolescence for adult performance and about possible biases in diagnostics caused by maturation related characteristics.
PURPOSE:
Focusing on players' speed abilities (SA) and technical skills (TS) with simultaneous consideration for anthropometric and relative age characteristics, this study analyzed the long-term prognostic relevance of a nationwide assessment conducted within the German soccer talent development program.
METHODS:
The prospective cohort study investigated N=14.178 talented players from the Under-12 age group born between 1993 and 1995. These players participated in a motor diagnostics (sprinting, agility, dribbling, ball control and shooting) carried out in 2004, 2005, or 2006, respectively. The measurement model comprised the five motor tests as well as three additional covariates (players' height, weight and relative age). As criterion for the adult performance level (APL) in the 2014/15 season, players were categorized as professional (N=89), semiprofessional (N=913), or amateur league players (N=13.176).
The prognostic relevance for each motor predictor was determined using one-way ANOVAs. Additionally, in a logistic regression model, APL was predicted by two latent variable factors (SA, TS) and the maturation related characteristics as covariates.
RESULTS:
Each motor predictor discriminated between the APL with small effect sizes (each $p<.001$; $\eta^2 \leq .02$). In the logistic regression model, TS (OR=6.72; $p<.001$) and SA (OR=4.58; $p<.05$) predicted the APL after controlling for height, weight and relative age. This multivariate threshold model explained R^2=24.8% of the APL variance.
CONCLUSIONS:
The study proved prognostic validity of motor predictors over a long-term period (≈10 years). However, the effect sizes indicated that the predictors explained only small proportions of the APL variance. Therefore, repeated measurements and further predictors (e.g. personality or cognitive performance factors) in a dynamic and multidimensional approach should supplement the diagnostics.
This study was funded by the German Soccer Association.

2799 Board #322 June 3, 11:00 AM - 12:30 PM

Assessing Assistant Referees' Movement Patterns during the First Half of a Professional Soccer Season

Kevin Huet[1], Andy Bosak[2], Matt Hawkey[3]. *[1]Kennesaw State University, Kennesaw, GA. [2]Liberty University, Lynchburg, VA. [3]Teesside University, Middlesbrough, United Kingdom.* (Sponsor: James Schoffstall, FACSM)
Email: khuet18@gmail.com
(No relationships reported)

PURPOSE: To examine North American Professional Soccer assistant referees' (ARs) movement pattern differences between the first and second halves of games during the first half of a soccer season. METHODS: Forty-seven North American ARs were observed during the first half of the 2014 regular season. Movement patterns were analyzed by a 2-D camera system and transferred from raw data to readily usable data. Standing, walking, jogging, and High-intensity Running (HIR) movement patterns (ie. running and sprinting) were assessed (ie. total distance covered and distance covered while moving in each speed zone) relative to each individual AR's predetermined maximum running speed. HIR has been reported in prior research as speeds greater than 13 km/h. Because this study utilized relative speed zones, our HIR values corresponded to speeds greater than 50% of the referee's max speed. Paired samples t-tests were utilized to assess all dependent variables. One-Way ANOVAs were conducted to analyze differences between first and second half values. RESULTS: ARs covered an average total distance of 6170.72 +/- 526.99m per game. Also, the ARs covered the majority (2854.25 +/- 327.56m, or 46.3%) of their total distance in the 21-50% ("jogging") of max speed range. Standing ($p<.001$) and walking ($p=.001$) increased in the second half, whereas jogging, running, and sprinting ($p=.018$) decreased. HIR accounted for 1212.31m, or 19.6%, of the total distance covered, yet decreased from 619.17m in the first half to 592.99m in the second half. Total distances covered were 3085.10m and 3086.31m in the first and second half, respectively. CONCLUSIONS: These professional North American Professional Soccer ARs appear to travel similar distances as their international counterparts. Despite the almost identical total distance covered in each half, it appears the referees covered less distances at higher speeds, suggesting the ARs may not have been physically recovered enough (i.e. due to demands of the first half, stress of traveling to the game, daily life stress, etc.) to meet the demands of the second half of the match. Since the primary role of the AR is to observe offside infractions, future research should be conducted in North America to determine where these ARs are specifically located on the sideline when an offside decision is to be decided.

2800 Board #323 June 3, 11:00 AM - 12:30 PM

Comparison Of Repeated Sprint Exercise And Square-wave Endurance Exercise In Young Soccer Players

Ruben Goebel[1], Zouhair Farhani[2], Wassim Moalla[3], Nabil Gmada[2], Ezedine Bouhlel[4], Monoem Haddad[1]. *[1]Qatar University, Doha, Qatar. [2]University of Jendouba, Kef, Tunisia. [3]University of Sfax, Sfax, Tunisia. [4]University of Sousse, Sousse, Tunisia.*
Email: ruben.goebel@qu.edu.qa
(No relationships reported)

Purpose: Performance optimization of athletes through exercise training interventions is a major task. The aim of this study was to compare the effectiveness of repeated sprint exercise (RSE) and the repeated square wave endurance exercise training (SWEET) to enhance the repeated sprint ability, power, and aerobic capacity in soccer players..
Methods: 22 male soccer players (age 19.9 ± 1.4 years) participated in the study. They were randomized in two groups: RSE group trained 3*6*(20+20m), with 4.30min of recovery between series and repetitions respectively and Sweet group trained 1min at 90% VO2peak, 4min at 50% VO2peak during 30 min both groups in addition to their traditional soccer training. The following parameters were measured before and after 7 training weeks (with 2 sessions per week): 10, 20, and 30 m linear sprints, blood lactate, repeated sprint performances (peak time, total time and fatigue index), the 5-jump performance, repeated sprint test (RST) and peak oxygen uptake (VO2peak).
Results: Linear sprint improved more after RSE than SWEET training ($p<0.01$).
RSE training showed larger improvements in the 5-jump scores and in RST. Peak power output and pedaling speed improved significantly higher through RSE training compared to the SWEET training ($p<0.01$). Delta blood lactate concentration was reduced after training. The reduction was more pronounced in SWEET group ($p<0.05$). Significant group x time interaction was found in the VO2peak ($p<0.001$), with SWEET showing larger improvement (4.9±1.3%) than RSE group (4.3± 1.5%).
Conclusion: The effects of repeated sprints on both aerobic and anaerobic metabolism shown in the present study are in agreement with other studies (Tonnessen et al. 2011, Serpiello et al. 2011). In agreement with the literature, the repeated sprint performances were increased significantly only in RSE group (Tonnessen et al. 2011). The improvement in power of the lower limbs and Vpeak for the RSE group reflects an enhancement in the ability to utilize the stored elastic energy and indirectly assists in the first phase of force-time curve initiated by the rate of force development in leg extensors within the RSE training group.
The present data showed that a specific training program based on RSE is superior to SWEET program to improve anaerobic performances in young soccer players.
